# Case report: Utilization of neutral density filters for densitometry analysis of dense corneal opacities

**DOI:** 10.1016/j.ajoc.2022.101672

**Published:** 2022-07-31

**Authors:** Akhil Meka, Cody Moezzi, Daniel Brocks

**Affiliations:** Department of Ophthalmology, BostonSight, Needham, MA, USA

**Keywords:** Corneal scar, Scheimpflug imaging, Scleral lens

## Abstract

**Purpose:**

This report describes the technique of utilizing a neutral density filter (NDF) during Scheimpflug imaging of a dense corneal opacity in order to increase data acquisition success and improve data reliability for densitometry analysis.

**Observations:**

A 49-year-old female with Steven-Johnson Syndrome secondary to sulfonamide use presented for routine follow up evaluation of her customized ocular surface prosthetic device (PD). Her ocular history was significant for mucous membrane grafting and limbal stem cell transplant in both eyes. The ocular surface examination of the left eye was notable for chronic dense neovascularization and scarring of the temporal and inferior cornea which extended into the visual axis. Scheimpflug imaging and densitometry analysis were performed in order to quantify the severity of the scar, however, there was significant difficulty in acquiring densitometry data. During a subsequent follow-up visit to monitor the scar, standardized room lighting and a neutral density filter were used to obtain reproducible and reliable imaging for densitometry analysis. The corneal scar was monitored over time using this standardized imaging protocol and by densitometry analysis minimal progression of the scar was evident, suggesting that recently documented significant vision loss in the left eye could not be attributed solely to changes in the scar.

**Conclusion and Importance:**

The use of a neutral density filter along with standardized ambient lighting conditions when performing Scheimpflug imaging may be necessary to reliably monitor densitometry progression of clinically severe corneal opacities.

## 1 Introduction

Corneal opacities, including corneal scars, are a common pathology associated with many ocular surface diseases. Opacities can occur at any histological layer of the cornea, including the epithelium, Bowman's membrane, stroma, Descemet's membrane and endothelium. Injury, chemical burns, infection, inflammation, and a wide spectrum of disease states can all cause corneal opacities. As described by Wilson et al., after an insult to the cornea occurs, recruitment of fibroblasts results in type 1 cytokine mediated rapid contraction and closure of the wound. The change in original collagen or proteoglycan structure alters the organized lattice structure leading to cornea opacification and scars.^1^

There is no agreed upon standardized method for clinically grading corneal opacities when examined by slit lamp biomicroscopy. In general ophthalmic or optometric practice, opacities, such as scars, are typically graded on size (utilizing the slit lamp adjustable knob for aperture height and adjacent ruler) and density. One example of a density scale utilized in clinical care is categorizing corneal opacities into three grades: nebular, macular and leucoma.^2^^,^^3^ This subjective qualitative system evaluates not only the density of the opacification but also the practitioner's ability to visualize iris architecture details, which may be obscured by the corneal pathology. Many clinicians instead prefer a numerical subjective system that is used to grade the corneal opacity from zero (clear cornea) to three (dense opacity), which relies on categorizing the opacity by both the density and the ability to visualize the underlying intraocular structures.^4^ Alternatively, practitioners may forego specific measurements of corneal opacities and instead utilize surrogate endpoints such as visual acuity to assess opacity severity and changes over time**.**

Quantitative objective analysis of many aspects of the cornea is necessary in clinical care as well as in research and is often accomplished utilizing Scheimpflug imaging (Pentacam, Oculus, Arlington, WA). This system is composed of a rotating Scheimpflug camera, from which a three-dimensional map of the cornea is created and multiple parameters can be assessed, including corneal densitometry analysis.^5^ The imaging device transmits light through the cornea, some of which will be scattered backwards if it is reflected off a corneal opacity; a software add-on then generates a map of the backscattered light throughout different areas of the cornea, called a corneal densitometry map (composed of densitometric values ranging from 0 to 100 standardized grayscale units). A value of zero units indicates no scar, while a value of 100 units indicates a dense scar. The program analyzes an area of 12 mm around the corneal apex, which is divided into four concentric zones for further densitometric evaluation.^6^

Densitometry is intended to be an objective quantitative method to evaluate corneal opacities, however, in our clinical experience, lack of a standardized in-office imaging protocol for the Scheimpflug camera operator when utilizing this technology may have drastic consequences on precision and the ability to reliably track opacity changes over time.^7^ In this report, we highlight the use of a standardized testing environment and present the technique of introducing a neutral density filter (NDF)^8^^,^^9^ (LEE Filters, Glazer's, Seattle, WA) to the Scheimpflug imaging system in order to increase data acquisition rates, improve data quality, and to re-calibrate the representative scale to accommodate the static and progressive densitometry analysis of dense corneal opacities.

## 2 Case report

A 49-year-old female with a remote past ocular history significant for Steven-Johnson Syndrome secondary to sulfonamide use presented for routine follow up consultation to reevaluate the function and fit of her customized ocular surface prosthetic device (PD), (PROSE, BostonSight, Needham, MA) in both eyes. Past ocular history was also significant for mucous membrane grafting and limbal stem cell transplant in both eyes, all completed greater than 10 years earlier.. Her current ocular medications in both eyes included loteprednol 0.5% twice a day, preservative free lubricating drops as needed and preservative free lubricating ointment at night. BCVA was 20/40 in the right eye and 20/30 in the left eye. On slit lamp examination, she had bilateral grossly keratinized lid margins, distichiasis and injected conjunctiva. There was peripheral pannus and mild inferotemporal corneal haze in the right eye. The left cornea was notable for chronic dense neovascularization and scarring of the temporal and inferior cornea which extended into the visual axis.

**Fig. 1:**
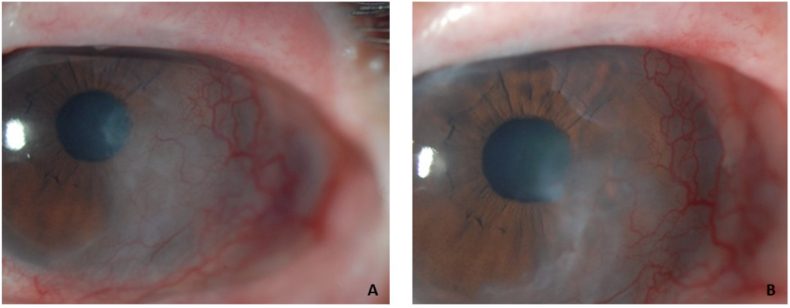
A: Slit lamp biomicroscopy photograph of left eye taken at Visit 1. Fig. 1B: Slit lamp biomicroscopy photograph of left eye taken at Visit 3. (Images courtesy of BostonSight, Needham MA).

**Fig. 2:**
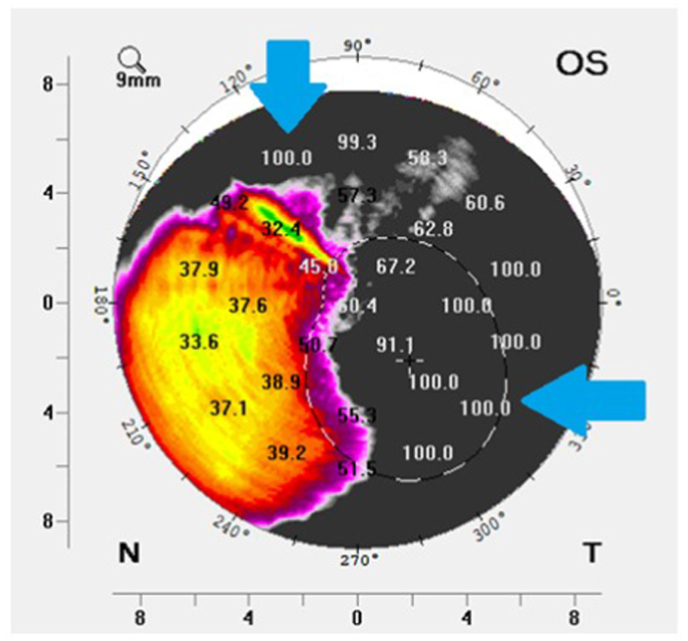
One of the densitometry data maps acquired at Visit 1, left eye. Significant portions of the map reach the maximum allowable value of 100 (blue arrows). (Image courtesy of BostonSight, Needham MA). (For interpretation of the references to colour in this figure legend, the reader is referred to the Web version of this article.)

The patient noted her primary ophthalmologist was considering further surgical intervention in the left eye including repeat limbal stem cell transplantation, however, her doctor wished to observe the left eye closely in the coming months to determine if some recent adjustments to and continued wear of her PD would mitigate further progression of the corneal neovascularization and scar. As part of the effort to closely follow any ocular surface changes over time, slit lamp photographs were taken (Fig. 1A). Additionally, Scheimpflug imaging densitometry data was acquired to quantitate the extent and severity of the cornea scar in the left eye, utilizing the following technique. Proparacaine was applied to the ocular surface to assist in patient comfort during imaging. Room lighting was standardized with the door being closed so no variable light pollution was entering the room from the hallway. Additionally, the lighting in the room was turned completely off. The Scheimpflug camera was set to acquire twenty-five images in 1 s at this and all subsequent visits. Despite excellent patient cooperation, steadiness and good ocular surface exposure, there was significant difficulty in acquiring densitometry data. Six attempts at acquiring densitometry data were undertaken with data acquired three times. Notable data flaws and registration issues were documented for all three of the scans that were able to acquire data, including missing data, hyper-reflectivity, limited scan diameter and erroneous posterior cornea surface registration (Table 1). All of the scans which acquired data had several areas that reached the upper limit of the representative densitometry scale (value of 100) (Table 1, Fig. 2). Average, standard deviation and range of the corneal scan data from visit 1 is summarized in Table 2.

**Table 1:** Data flaws with densitometry acquisition, left eye.

	missing data	hyper-reflectivity	limited scan diameter	erroneous posterior surface registration	reached densitometry scale upper limit	no data acquisition flaws
Visit 1						
Scan 1, no NDF	✓	✓	✓	✓	✓	
Scan 2, no NDF		✓	✓	✓	✓	
Scan 3, no NDF		✓	✓		✓	
Visit 2						
Scan 1, no NDF		✓	✓	✓	✓	
Scan 2, with NDF						
Scan 3, with NDF						
Scan 4, with NDF						
Visit 3						
Scan 1, with NDF						
Scan 2, with NDF						
Scan 3, with NDF						
Scan 4, with NDF						

NDF = neutral density filter.

**Table 2:** Corneal densitometry average, standard deviation and range by visit, left eye.

	Corneal densitometry (grayscale units)		
	Total (full thickness, 0–12 mm)	Visual axis (full thickness, 0–2mm)	Visual axis (full thickness, 2–6mm)
Average			
Visit 1 (3 scans)	40.87	34.73	37.83
Visit 2 (3 scans)	16.13	11.40	13.10
Visit 3 (4 scans)	17.00	12.03	13.63
Standard deviation			
Visit 1 (3 scans)	1.37	4.17	1.62
Visit 2 (3 scans)	0.55	0.17	0.26
Visit 3 (4 scans)	0.36	0.82	0.76
Range			
Visit 1 (3 scans)	2.50	8.30	2.90
Visit 2 (3 scans)	1.00	0.30	0.50
Visit 3 (4 scans)	0.80	1.90	1.80

Visit 1 = WITHOUT neutral density filter.

Visit 2 = WITH neutral density filter.

Visit 3 = WITH neutral density filter.

The patient presented two months later for follow up examination to re-evaluate the left eye for any progressive ocular surface changes. BCVA was stable at 20/30–2 in the left eye. There were no changes in the medication regimen and slit lamp examination of the left eye was stable.

The risk of progressive scarring and neovascularization continued to be of concern in the left eye. To continue to closely observe the left eye for progressive changes, Scheimpflug imaging was repeated to acquire densitometry data. One scan was attempted duplicating the scan technique and conditions from visit 1. Once again, data flaws, poor quality images and data reaching the maximum allowable value (100) resulted. To attempt to address these persistent challenges and to try to obtain reproducible data suitable for tracking and future comparison, a novel technique was undertaken utilizing an NDF.

**Fig. 3:**
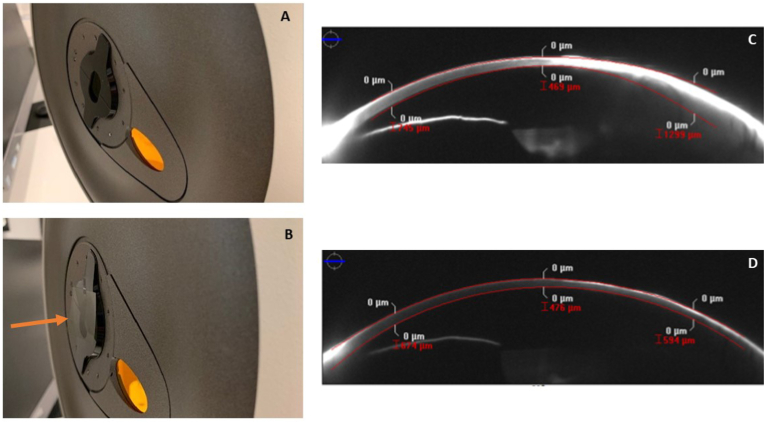
A: Scheimpflug camera without 0.6 NDF, B Scheimpflug camera with 0.6 NDF in position (orange arrow), C: Scheimpflug image taken without NDF, D: Scheimpflug image taken with NDF. Note the reduction in reflectivity and proper posterior corneal surface registration with NDF in place. NDF: neutral density filter. (Image courtesy of BostonSight, Needham MA). (For interpretation of the references to colour in this figure legend, the reader is referred to the Web version of this article.)

As before, room lighting was standardized with the door being closed so no variable light pollution was entering the room from the hallway. Lighting in the room was once again turned completely off. The patient received proparacaine eye drops. A 0.6 NDF was carefully and gently taped over the Scheimpflug imaging device light source to reduce light intensity in all wavelengths in order to minimize hyper-reflectivity (which was believed to be causing poor registration and lack of data acquisition) and to re-calibrate the densitometry scale (Fig. 3). Three scans were then taken of the left eye, with data acquired in each scan. All scans had no notable data flaws or registration issues. None of the scans reached the upper limit of the representative densitometry scale (Table 1). Average, standard deviation and range of the corneal scan data for visit 2 are provided in Table 2. Given the quality and reproducibility of these images, the determination was made to have these new images represent the baseline data and for the opacity to be tracked moving forward utilizing this new standardized imaging technique and protocol.

Five months later, the patient returned for a follow up examination. The patient noted worsened vision in the left eye over the last several months. She raised concerns by her primary ophthalmologist that there had been interim progression of the cornea scar to account for the vision loss. BCVA was notably reduced in the left eye to 20/100. There had been no changes in the medication regimen. The slit lamp examination again appeared without significant change, though there was a question of possible minimally increased haze in the paracentral portion of the scar when careful examination was undertaken of slit lamp photographs from Visit 1 compared to Visit 3 (Fig. 1A and B).

**Fig. 4:**
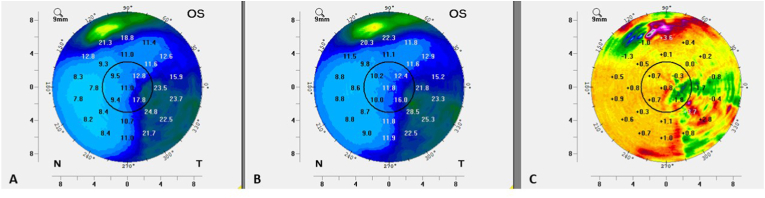
Average (full thickness cornea) densitometry readings. A: Visit 2, B: Visit 3, C: Change in average densitometry B-A, with a positive value signifying worsened opacification. Note the central black solid line circle in each image demarcates the central 3mm of the cornea.

Scheimpflug imaging was once again repeated in order to evaluate densitometry for any objective progressive opacification of the corneal scars since the prior examination. The patient received proparacaine eye drops in the left eye. Room lighting was standardized to match prior environment conditions-the door to the imaging room was closed, the lighting in the room was turned off and a 0.6 NDF was placed over the Scheimpflug imaging device light source. Four scans were taken of the left eye, with data acquisition successful in all scans. All scans had no notable data flaws or registration issues. None of the scans reached the upper limit of the representative densitometry scale (Table 1). Average, standard deviation and range of the corneal scan data for visit 3 are provided in Table 2. Utilizing the “Compare 2 Exams” functionality on the Scheimpflug imaging device, average corneal densitometry data maps were compared from a Visit 2 scan to a Visit 3 scan (Fig. 4). Special attention was given to the data in the central 3mm of the cornea average densitometry maps to ascertain if any significant progression in opacification was evident visit to visit in the visual axis. Minor fluctuations (both up and down) were evident in various areas within the 3mm zone. In addition, focused review of the average change in densitometry in the visual axis from visit 2 to 3 in the 0–2mm and 2–6mm corneal zones was undertaken and revealed a modest average increased densitometry in both zones (Table 2). Average densitometry increased in the 0–2mm zone by 0.63 grayscale units and in the 2–6mm zone by 0.53 grayscale units. When taken in toto, the data was interpreted to indicate at most a minimal progression of the central corneal scar, within the standard bounds of the visual axis, had taken place over the 5 month follow up period. Densitometry analysis alone did not seem to reveal a significant progressive opacification that could be well correlated to the dramatic vision reduction in the left eye from Visit 2 (20/30–2) to Visit 3 (20/100). Upon further evaluation and return visit to the referring provider, including comprehensive evaluation of the lens and posterior pole, we concluded the vision reduction was most likely relatable to not only the minimal corneal opacity changes but more so due to progressive posterior subcapsular cataract, which was not evident on prior history or examinations.

## 3 Discussion

Densitometry provides the potential for an objective methodology for evaluating and tracking corneal opacity severity and changes over time.^10^ Current subjective grading systems used in clinical practice are problematic. For instance, the subjective grading of slit lamp photographs or subjective grading of corneal opacities during slit lamp examination raises inherent obvious concerns of precision and accuracy. Intra-observer and inter-observer variability is of high risk, as has been reported with other slit lamp exam parameters, such as measuring corneal ulcers.^11^ Additionally, opacities on slit lamp examination and slit lamp photographs can appear dramatically different depending on the operator's technique due to there being no standardization of light intensity or slit lamp arm angle when evaluating corneal opacities. With Scheimpflug imaging, these parameters are fixed, however, additional external factors, such as environmental lighting conditions, need to be controlled to optimize the utility of densitometry imaging. Other modalities, such as OCT have been attempted for this purpose in the past, however, to date, there exists no standardized, validated, integrated OCT opacity analysis system with which to analyze the imaged cornea.^2^

Dense scars, however, can pose additional obstacles to acquiring densitometry data. As described in the visits highlighted in this case report, all images taken without an NDF resulted in hyper-reflectivity off the corneal opacities which we postulate resulted in significant imaging artifact and scan acquisition failures. As described, without the use of an NDF in this case, it took six attempts to successfully acquire three scans during Visit 1.

As illustrated in this case report, even when scans were successfully acquired without an NDF, the hyper-reflectivity off the dense scar resulted in poor quality data with notable flaws, such as missing data, limited scan diameter data acquisition, and erroneous registration of the posterior corneal surface. These notable data flaws were present in every instance when the cornea was imaged without the NDF, including all three scans acquired in Visit 1 and the first scan acquired in Visit 2 (the only scan done without an NDF during Visit 2). This underscores that densitometry imaging of dense scars may not only be prone to acquisition failure but also data errors.

The potential utility of the addition of an NDF for acquiring densitometry analysis of dense scars is five-fold; reduce hyper-reflectivity, increase data acquisition success, reduce data flaws (improve data quality), increase data precision, and re-calibrate the densitometry scale to accommodate ongoing monitoring of dense scars. As detailed in Table 1, all scans attempted with an NDF in place were successfully acquired, had no notable data flaws and did not max out the relative densitometry scale (no densitometry maximum values of 100 were reached). Additionally, as documented in Table 2, the standard deviation and range of multiple scans taken in succession at an individual visit were dramatically reduced from Visit 1 (no NDF) in comparison to Visit 2 or 3 (images done with NDF), underscoring an improved precision with the use of an NDF.

An NDF is an optical filter that absorbs light of all wavelengths equally, causing an overall reduction in light intensity throughout the visible light spectrum. This will limit the risk of over-exposure (or hyper-reflectivity) with dense scars imaged with the Scheimpflug camera. It is important to note that there are a variety of NDF strengths (termed “optical density”) and for this case we chose an optical density of 0.6, a selection based on our prior experience.^8^^,^^9^ From our clinical experience, depending on the severity of the scar, we have found the options of no filter, or an optical density of 0.3 or 0.6 to be appropriate options to maintain in stock for clinical use. In most instances, a mild scar will require no NDF, a moderate scar may require no NDF or a 0.3 filter, while a dense scar may require the higher strength 0.6 filter.^12^ If too strong of a filter is used, only a faint Scheimpflug image of the cornea will be visible and densitometry values will hover on or close to zero on post-scan image review, which also will be un-useable data to analyze or track over time. It is important of course, to utilize the same filter at each subsequent visit for an individual patient in order to have valid comparison over time. With the standardization of lighting for all patients and the individualized standardization of the use of NDFs for each individual patient, the utility of densitometry for use in dense scar analysis could be dramatically improved.

Future studies most certainly are needed to further assess, standardize and optimize the use of NDFs. Determining a single existing filter strength (or developing one) that may be applicable to most cases would be optimal. Determining conversion factors to comparatively assess images taken with different filters would as well be advantageous. Integration of these filters within the hardware and software of the Scheimpflug imaging device would be of great benefit for standardization.. Of great importance, in order to make meaningful use of densitometry data, further research is needed into elucidating what constitutes a minimal clinically important difference (MCID) in densitometry from one visit to the next, akin to the research produced for other important ophthalmic parameters such as Ocular Surface Disease Index (OSDI).^13^ As described in this case report, the average densitometry increased in the 0–2mm zone by 0.63 and in the 2–6mm zone by 0.53 grayscale units from Visit 2 toVisit 3, but the potential clinical significance of this degree of change is currently not well understood.

## 4 Conclusion

Evaluating corneal opacities for progressive changes over time is typically challenging in both clinical practice and research when the documentation is based on subjective observations. A common approach is to use a 0 to 3 subjective grading system^4^ which can result in significant loss of intra-observer and inter-observer precision and accuracy.

Scheimpflug imaging provides a potential objective quantitative solution to standardize the grading of corneal opacities through the use of densitometry data analysis. However, this case report highlights several potential obstacles to reliability, particularly when imaging dense corneal scars. Most notably, the technology depends on standardized ambient lighting conditions as well as the consideration of the use of an NDF for imaging dense opacities in order to improve data acquisition rates, improve scan quality, reduce data errors, improve data precision and to re-calibrate the densitometry scale. Future clinical studies are necessary not only to further investigate whether densitometry can reliably provide reproducible data to accurately track progression or regression of corneal opacities but also to fully elucidate the possible standard role an NDF could have on the imaging process.

## Data availability

N/A.

## Funding

No Funding or grant support

## Authorship

All authors attest they meet the current ICMJE criteria for authorship.

## Patient consent to publication

Written informed consent regarding risks and benefits of prosthetic replacement of the ocular surface ecosystem treatment was obtained from the patient. Consent to publish the case report was not obtained, as this case report does not contain any identifiable health information or identifiable personal information that could lead to the identification of the patient. All guidelines were followed to ensure HIPAA compliance, and we adhered to the Declaration of Helsinki, as well as applicable federal and state laws.

## Declaration of competing interest

The authors report no financial conflicts of interest in this work. Daniel Brocks is a salaried clinical employee of BostonSight, Needham, MA. None of the authors have a propriety or financial interest in PROSE (BostonSight, Needham MA) or the prosthetic devices used in PROSE treatment.

## References

[1] Wilson S.L., El Haj A.J., Yang Y. (2012). Control of scar tissue formation in the cornea: strategies in clinical and corneal tissue engineering. J Funct Biomater.

[2] Rose J.S., Eldrina J., Joshua A. (2018). Objective quantification of corneal haziness using anterior segment optical coherence tomography. J Curr Ophthalmol.

[3] Parsons J.H. (2011). Parson's Diseases of the Eye.

[4] Shimizu E., Yamaguchi T., Tsubota K., Shimazaki J. (2019). Corneal higher-order aberrations in eyes with corneal scar after traumatic perforation. Eye Contact Lens.

[5] Motlagh M.N., Moshirfar M., Murri M.S. (2019). Pentacam(R) corneal tomography for screening of refractive surgery candidates: a review of the literature, Part I. Med Hypothesis, Discov Innovation (MEHDI) Ophthalmol.

[6] OCULUS Pentacam AXL Wave (2020).

[7] Ni Dhubhghaill S., Rozema J.J., Jongenelen S., Ruiz Hidalgo I., Zakaria N., Tassignon M.J. (2014). Normative values for corneal densitometry analysis by Scheimpflug optical assessment. Invest Ophthalmol Vis Sci.

[8] A Beginner's Guide to Neutral Density Filters. Australia: URTH Magazine. Available from: https://urth.co/magazine/neutral-density-filter-explained. Accessed 2022.

[9] Newton M. ND Filters – in Depth Guide for Beginners. The School of Photography. Available from: https://www.theschoolofphotography.com/tutorials/nd-filters-in-depth-guide-for-beginners. Accessed 2022.

[10] L M.J., Greenstein S.A., Gelles J.D., Hersh P.S. (2020). Corneal haze after transepithelial collagen cross-linking for keratoconus: a Scheimpflug densitometry analysis. Cornea.

[11] Patel T.P., Prajna N.V., Farsiu S. (2018). Novel image-based analysis for reduction of clinician-dependent variability in measurement of the corneal ulcer size. Cornea.

[12] Vorenkamp T. (2017). https://www.bhphotovideo.com/explora/photography/hands-review/guide-neutral-density-filters.

[13] Miller K.L., Walt J.G., Mink D.R. (2010). Minimal clinically important difference for the ocular surface disease index. Arch Ophthalmol.

